# A Case of Pulmonary Artery Endarteritis With Patent Ductus Arteriosus

**DOI:** 10.7759/cureus.76696

**Published:** 2024-12-31

**Authors:** Son H Dam, Quynh N Ninh, Huu C Nguyen, Dat T Pham

**Affiliations:** 1 Department of Cardiac and Thoracic Surgery, Cardiovascular Center, E Hospital, Hanoi, VNM; 2 Department of Adult Cardiovascular Disease, Cardiovascular Center, E Hospital, Hanoi, VNM; 3 School of Medicine and Pharmacy, Vietnam National University, Hanoi, VNM

**Keywords:** congenital heart disease, endocarditis, patent ductus arteriosus (pda), pulmonary artery endarteritis, pulmonary embolism, staph aureus endocarditis

## Abstract

Right-sided heart endocarditis contributes to a small portion of infective endocarditis (IE) cases. Right-sided endocarditis related to undiagnosed congenital heart defects is even more scarce. Prompt diagnosis by transthoracic echocardiography (TTE), aggressive antibiotics from the beginning, and surgical removal of the vegetation for IE help prevent the risk of multi-organ failure and fatal pulmonary embolism. This is a case of a 38-year-old female, with normal medical history and recent vaginal birth at a district hospital four months ago. She was admitted to the hospital because of fever and continuous shortness of breath one month before admission. TTE detected vegetation (10x23 mm) on the left pulmonary artery and patent ductus arteriosus. A CT scan showed several abnormal mobile mixed-echo masses in the left pulmonary artery and partial pulmonary embolism. The patient had surgery to remove the vegetation and close the patent ductus arteriosus. The patient was stable after surgery and discharged two weeks after surgery. She continued two-week oral antibiotics at home and made a follow-up appointment one month later. Pulmonary artery endarteritis associated with patent ductus arteriosus is a rare lesion and has a high risk of death. The diagnosis should be considered in any febrile, septic patient with congenital heart disease. Removing vegetation and aggressive antibiotic treatment should be performed together to improve the outcome.

## Introduction

The ductus arteriosus is an opening between the aortic and main pulmonary artery, which usually closes after birth along with a decrease in prostaglandin E2 concentration and an increase in arterial oxygen concentration. Patent ductus arteriosus (PDA) is a heart defect when the ductus arteriosus stays open or does not close completely. PDA causes a left-to-right shunt and, if untreated, results in pulmonary overcirculation. Besides atrial septal defect and ventricular septal defect, PDA is the third most common adult congenital heart disease (ACHD) [1]. Infective endocarditis (IE) in ACHD is a rare condition with a prevalence ranging from 0.9 to 1.33 cases per 1000 patients [2] and a mortality rate of 9.18% [3]. In particular, PDA‐associated infective endarteritis (PDA-IE) has a high mortality rate (approximately 45%) according to the data from the 1960s [4]. In recent years, the mortality rate of PDA-IE has decreased due to the advancement of multiple diagnostic methods (echocardiography, vascular CT scan, vascular MRI), proper treatment with aggressive use of antibiotics, and timely intervention with surgical closure of PDA. Particularly, the development of thoracic endoscopic surgery in terms of congenital heart disease allows to reduce the critical care period and facilitates patient amelioration.

## Case presentation

A 38-year-old female was admitted to the hospital due to general malaise and intermittent fever. She gave vaginal birth to a baby boy four months ago in a local hospital. Within one month, she felt febrile with a temperature ranging from 38.5 to 39 °C and deteriorating shortness of breath. She tried to use over-the-counter antibiotics but with only minor improvement and is still febrile. These days, she felt more tired. She had lost 5 kg in one month. Then, she came to our hospital. On admission, her blood pressure was 120/60 mm Hg, with a regular heart rate of 98 beats per minute, a respiratory rate of 24 times per minute, and an axillary temperature of 38.2 °C. Physical examination revealed conjunctival pallor without obvious signs of infective endocarditis, such as Osler's nodes and Janeway lesions. Jugular venous pressure was not elevated. On auscultation, there was a 3/6 grade continuous murmur at the pulmonary area without radiation and another 4/6 grade (according to the Levine scale) holosystolic murmur in the upper left sternal border. The findings of the lung examination were within normal limits. An abdominal examination revealed no organomegaly. There were no signs of cyanosis or clubbing.

**Figure 1:**
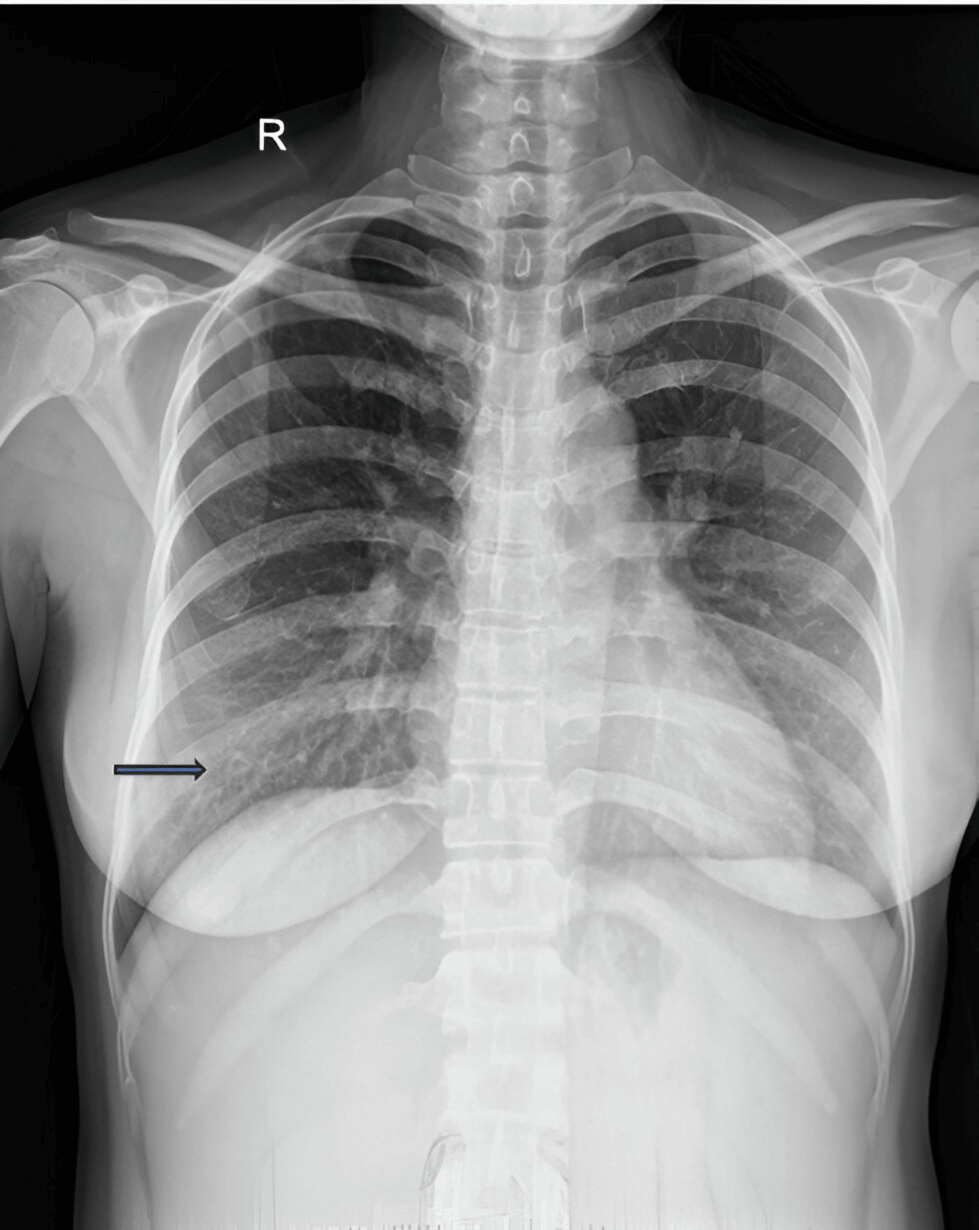
Chest X-ray upon admission The arrow points to small shape cavities suspicious of micro-abcesseses.

Laboratory data indicated anemia, her hemoglobin level was 86 g/dL, her leukocyte count was 11.130 per cubic mm, and her platelet count was 294 per cubic mm. The pro-calcitonin level drawing on the admission day was 2.47 (normal index: < 0.046 ng/mL). The serum albumin concentration was 30.9 g/dL. The liver and kidney functions are listed in Table 1. The 12-lead electrocardiography showed sinus tachycardia. Chest X-ray revealed a nearly normal cardiac silhouette, but some small opacities in the right lower lobe might be suspected micro-abcesses with overt circles and pus inside (Figure 1).

**Table 1:** Laboratory test results at admission

Name	Patient’s result	Normal range	Unit
Hemoglobin	86	12.5-14.5	g/dL
Leukocyte	11.13	4-10	x 10^3^/ml
% Neurophil	66	41-74	%
Platelet	294	150-400	x 10^3^/mL
AST (Aspartate aminotransferase)	29	< 35	U/L
ALT (Alanine aminotransferase)	52	<35	Mcmol/L
Creatinin	60.9	58-96	Mcmol/L
Albumin	30.9	35-52	g/L
Glucose	3.6	3.9-5.6	Mmol/L

**Figure 2:**
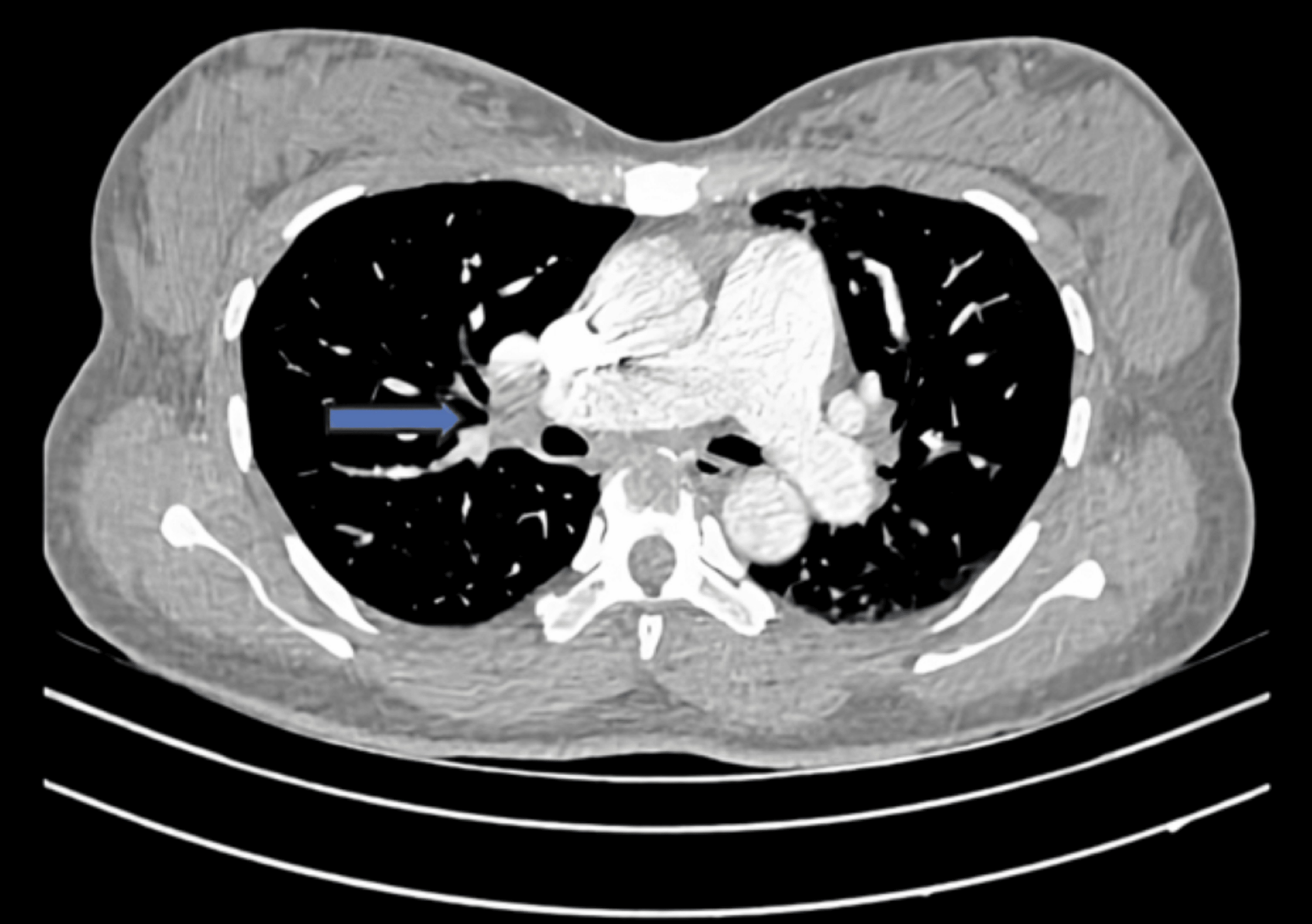
Partial pulmonary embolism (PE)

Pulmonary CT angiography revealed a partial embolism in the main and left pulmonary artery (Figure 2), and it was a partial embolism from the large vessels to the distant branches, which suggested scattered septic emboli.

**Figure 3:**
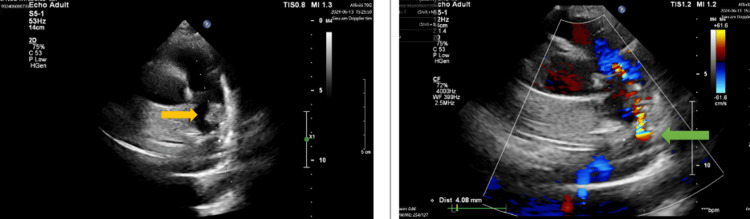
Patent ductus arteriosus (PDA) with large vegetation at the pulmonary ending

**Figure 4:**
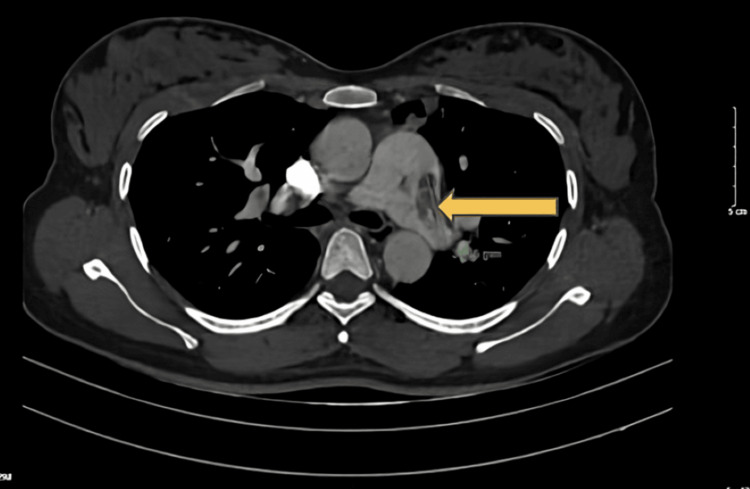
Vegetation on a plain chest CT scan

Transthoracic echocardiography (TTE) confirmed a PDA with continuous left-to-right shunt, size 5.8 mm in the left pulmonary input and 7.6 mm in the aortic side. Multiple vegetations (with the largest and most highly mobile vegetation sized 10 x 23 mm) were attached to the wall of the left main pulmonary artery (Figure 3). Right and left ventricular sizes and function were within normal range, without other significant valve diseases. The estimated pulmonary pressure on TTE was 40 mmHg. The plain CT confirms micro-abscesses and vegetation attaching to the left pulmonary artery (Figure 4).

The patient was diagnosed with a left-to-right shunt PDA with intermittent fevers, probably due to suspected IE (based on the modified Duke criteria). Peripheral blood samples from three separate sites (right arm, left arm, right leg) were withdrawn for bacterial culture and minimum inhibitory concentration test to guide antibiotic treatment. Subsequent investigations confirmed that Staphylococcus aureus had been grown, and the patient was immediately placed on a combined intravenous antibiotic regimen.

**Figure 5:**
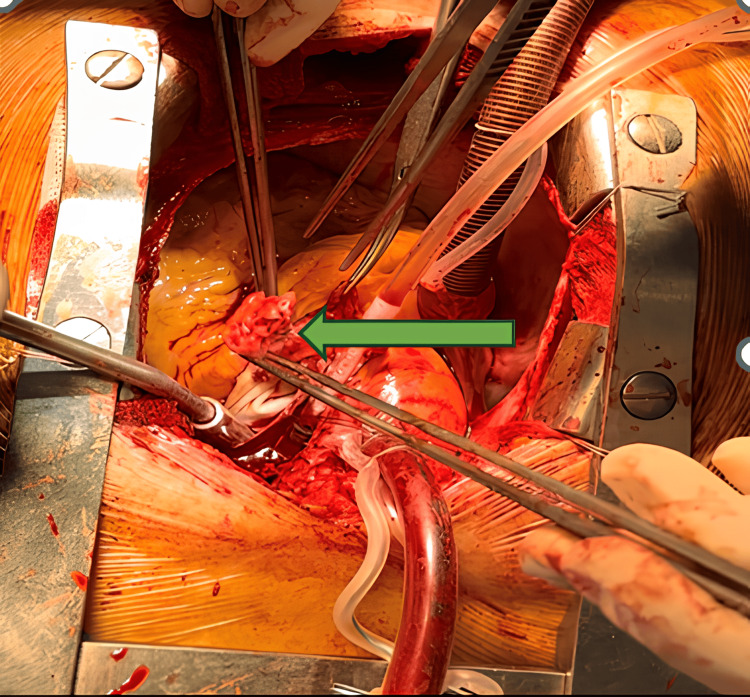
Intra-operation image of extracting the vegetation

At first, the patient was administered meropenem 1,000 mg intravenously every 12 hours and levofloxacin 500 mg every 24 hours while awaiting blood culture reports. She was not prescribed any anti-arrhythmias or anti-thrombotics during her hospital stay. A small dose of furosemide (20 mg orally) was prescribed due to the concern about pulmonary overcirculation and titrated based on the patient's response. Blood cultures later revealed the growth of Staphylococcus aureus. Later the antibiotics were changed to meropenem 1000 mg every eight hours, levofloxacin 500 mg every 24 hours, and vancomycin 1,000 mg every 12 hours. Five days after admission, the patient was afebrile. After 14 days of hospitalization, the patient underwent surgery for closure of the PDA and resection of the vegetation (Figure 5). During the operation, the native valve morphology appeared relatively thin in appearance with normal leaflet motion. There were multiple vegetations in the main trunk and left pulmonary artery related to the input of the PDA. The resected specimen was sent to culture and came back with a negative result one week later.

After 28 days, the patient had improved and was hemodynamically stable. The post-operative TTE showed no shunt flow or vegetation, and the estimated pulmonary pressure was 31 mmHg. The patient was discharged with a prescribed two-week oral antibiotic: amoxicillin x 2 g/day and levofloxacin 500 mg/day.

## Discussion

PDA in adults can lead to a wide range of hemodynamic abnormalities and clinical symptoms, including heart failure, pulmonary hypertension, and arrhythmia depending on the volume of left-to-right shunt blood flow through the ductus arteriosus. IE, one of the most serious complications of PDA, was previously rather common [1]. However, it has become scarce in recent years (0.24 per 1000 person‐years [1], compared with the proportion of 1.33 cases per 1,000 persons per year in the general ACHD population [2]. There has been continued progress in the diagnosis and management of IE over the years. However, the recognition of this entity remains a challenge due to its rarity and various clinical presentation.

These patients were often admitted with atypical clinical features. The most common symptom reported in multiple cases was persistent prolonged fever, often from two to four weeks with prior antibiotics before tertiary care [5,6]. Others presented with dyspnea, chest pain, weight loss, lethargy, and uncommonly hemoptysis as reported by one study of patients diagnosed with septic pulmonary embolism [7]. A wide spectrum of risk factors ranging from intravenous drug addiction, cardiac implantable electronic devices (CIED), central lines, immunosuppressive therapy, and several medical procedures such as transcatheter procedures have been associated with PDA-IE [8]. In this case, we presumed that the etiology of infection might be from the improper hygienic vaginal procedure during the child-bearing four months ago.

In centers with expertise in cardiovascular and ACHD imaging, TTE provides fundamental and reliable morphological and functional information for a correct diagnosis, for guiding the management strategy, and for monitoring treatment response. Variable and off-axis unconventional echo views may be effective in clearly visualizing all the structures in the RVOT and the first branches of the pulmonary trunk. Pulmonary artery and PDA visualization may be limited by its anterior position in the chest, appearing in the far field from the thoracic approach. Due to its infrequency, endocarditis involving this area is missed in real-world scenarios, especially in patients with no known clue of ACHD history, like in this case. Careful inspection of the parasternal short-axis view is the most useful, in addition to the subcostal view providing a diagnosis in 87%-91% [9]. In case TTE is insufficient but still highly suspicious, transoesophageal echo plays an important role, especially in accurately ruling out the involvement of other cardiac structures, such as valve dysfunctions and subvalvular apparatus, mechanical complications, or intra-cardiac shunts. Some preferred views for visualizing a PDA on a transesophageal echocardiogram are the cranial position and the high esophageal aortic arch long-axis view.

It is suggested that adjunctive imaging techniques should be utilized to provide comprehensive information for prognostic and management. High-resolution computed tomography diagnoses pulmonary complications (PE, abscess, infarction, effusion) and large (>10 mm, mobile) vegetations associated with embolization [10]. PE has been attributed to septic emboli or a jet hitting the wall of the PA. Some data reported that septic pulmonary emboli occur in 24.5%-75% of patients with right heart side endacteritis [11,12]. Some evidence proved that staphylococcal endocarditis is also a risk factor for embolization, which should be noticed because the incidence of S. aureus IE is increasing [13]. Furthermore, CT angiography and cardiac magnetic resonance imaging provide high-resolution images and attribute for the comprehensive diagnosis of concomitant congenital malformations, septic pulmonary infarction, pseudo-aneurysm, fistulas, valve perforation, abscess, and valvular dehiscence [14].

Streptococcus viridians (19/35 cases) and Staphylococcal (9/35 cases) are the most common microorganisms found in patients with PDA [12]. However, negative culture IE can occur in about 10% of all cases [12]. In reality, the prevalence of negative blood culture might be higher due to misdiagnosis and over-the-counter antibiotic use before admission, which is still popular in developing countries, such as Vietnam. Fortunately, a favorable outcome was observed in patients who grew Staphylococcus or S. viridians. Poor prognosis is related to fungal infection, poly-microbial, and culture-negative endocarditis [15,16]. In this patient, we decided to use a combined broad-spectrum antibiotic (meropenem and levofloxacin) before having a blood culture result due to the concern about antibiotic resistance and our patient's condition. Then, the blood culture was positive for S. aerius and guided for antibiotic management, which contributed significantly to the septic clearance.

Current European Society of Cardiology (ESC) guidelines recommend the closure of PDA in cases of left ventricular volume overload or pulmonary artery hypertension due to a significant left-to-right shunt flow through PDA [17]. There remains controversy regarding the closure of “silent” PDA without significant hemodynamic changes [18]. Regarding PDA‐IE, regular PDA closure solely for its primary prevention has not been recommended due to the concern of a rare occurrence of PDA-IE and the cost-effectiveness of the procedure [17]. However, it remains a risk factor for infective endocarditis, typically in developing countries [19]. Evidence supports that PDA-IE patients should have surgery because the infective endarteritis associated with PDA can cause severe complications such as organ failure due to septic emboli, fatal arrhythmias, and neurological problems, such as a stroke or mycotic aneurysm [6,7]. In the present case, surgical PDA ligation was implemented after careful consideration to prevent future embolism and secondary PDA-IE.

## Conclusions

This case reports dangerous IE complicated by undiagnostic PDA. Due to its rarity and atypical symptoms, PDA-IE is still a challenge in terms of timely diagnosis and proper management. Echocardiography is a fundamental method of diagnosis. Careful looks beyond the valves might help in the suspicion of endocarditis. Early aggressive antibiotic regimens are important to prevent fatal outcomes. Surgical PDA closure should be considered for the prevention of secondary complications.
